# Current approaches for the regeneration and reconstruction of ocular surface in dry eye

**DOI:** 10.3389/fmed.2022.885780

**Published:** 2022-09-23

**Authors:** Vimal Kishor Singh, Pallavi Sharma, Uttkarsh Kumar Sharma Vaksh, Ramesh Chandra

**Affiliations:** ^1^Department of Biomedical Engineering, Amity School of Engineering and Technology, Amity University, Noida, Uttar Pradesh, India; ^2^Tissue Engineering and Regenerative Medicine Research Lab, Department of Biomedical Engineering, Amity School of Engineering and Technology, Amity University, Noida, Uttar Pradesh, India; ^3^Tissue Engineering and Regenerative Medicine Research Lab, Department of Biomedical Engineering, Amity School of Engineering and Technology, Amity University, Gurgaon, Haryana, India; ^4^Institute of Nanomedical Sciences, University of Delhi, Delhi, India

**Keywords:** dry eyes, tear film, lacrimal gland, biomaterials, scaffolds, organoids, stem cells, Sjögren's syndrome

## Abstract

Significant research revealed the preocular tear film composition and regulations that remain vital for maintaining Ocular surface functional integrity. Inflammation triggered by many factors is the hallmark of Ocular surface disorders or dry eyes syndrome (DES). The tear deficiencies may lead to ocular surface desiccation, corneal ulceration and/or perforation, higher rates of infectious disease, and the risk of severe visual impairment and blindness. Clinical management remains largely supportive, palliative, and frequent, lifelong use of different lubricating agents. However, few advancements such as punctal plugs, non-steroidal anti-inflammatory drugs, and salivary gland autografts are of limited use. Cell-based therapies, tissue engineering, and regenerative medicine, have recently evolved as long-term cures for many diseases, including ophthalmic diseases. The present article focuses on the different regenerative medicine and reconstruction/bioengineered lacrimal gland formation strategies reported so far, along with their limiting factors and feasibility as an effective cure in future.

## Introduction

**Figure 1:**
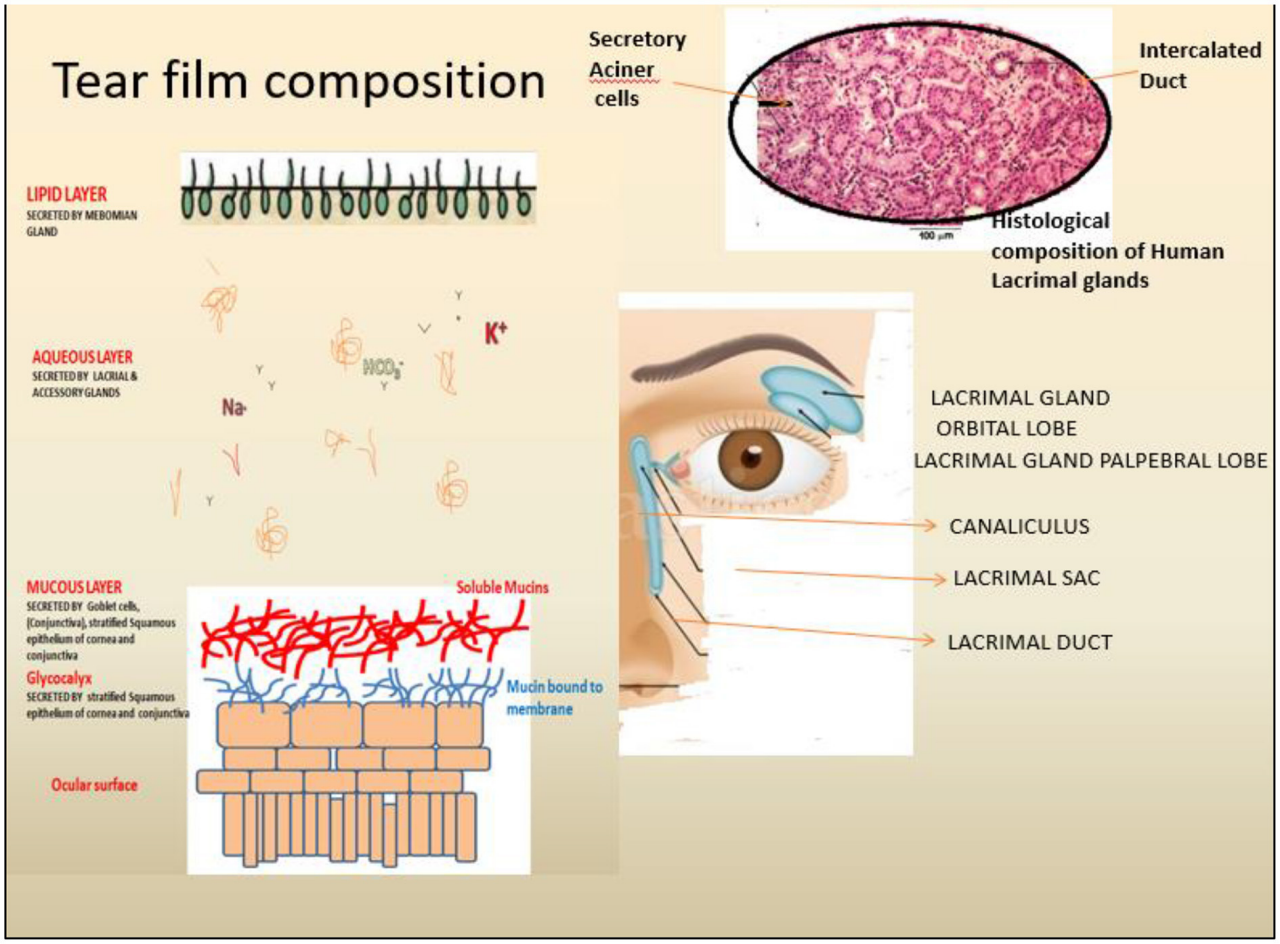
Anatomical and physiological depiction of the human lacrimal gland and associated components. Tears are secreted by Lacrimal gland function units, including the lacrimal gland (LG) (Blue), the ocular surface (cornea, conjunctiva, and the meibomian gland) and the associated sensory and motor nerves. The tear film comprises three significant layers, i.e., Outer lipid, middle Aqueous and inner mucin-rich layers secreted by (Left) secreted by the meibomian gland, lacrimal gland and ocular surface goblet cells as depicted.

The human ocular surface health essentially depends on a stable thin layer of tears (tear film) acting as a protective barrier against the external environment, thus keeping the surface moistened and maintaining ocular epithelial surface homeostasis. Multiple components are responsible for the regular function of the Lacrimal Functional Unit (LFU), including the lacrimal gland, the eye surface (cornea, conjunctiva, and meibomian gland), and related sensor and motor neurons (1–4). The LFU regularly create tear film to maintain corneal transparency, and image quality projected onto the retina because it is the central regulator of the secretion of the major components of the tear film. Aqueous layer secretions, as well as basal and reflex tearing in response to sensations from the outer surface, are controlled by the outer lipid, middle aqueous, and inner mucin layers of the mammalian tear film (Figure 1). These LG are found beneath the orbital's upper temporal compartment in the lacrimal fossa in orbital cavities (4). Many factors, such as advancing age, autoimmune diseases, orbital radiotherapeutics, and a low androgen pool, can cause Lacrimal gland dysfunction, commonly referred to as Dry Eye Syndrome (DES) or Dry Eye Disorders (DEDs) (Table 1: Causing agents). The dry eye syndrome (DES) is defined as “a multifactorial disease of the ocular surface characterized by a loss of homeostasis of the tear film and accompanied by ocular symptoms, in which tear film instability and hyperosmolarity, ocular surface inflammation and damage, and neurosensory abnormalities play etiological roles” (at TFOS/DEWS II) (8). Different types of DES have been defined and classified based on the causing agents (9). Other complicated factors such as ·Non-aqueous Sjögren's deficiency (e.g., age-related) ·Dry eyes are caused by a lack of aqueous humor, such as Sjögren's syndrome. The global prevalence of dry eye disorders (DEDs) is between 11 and 22%, but it is estimated to be between 18.4 and 20% in the Indian subcontinent.

**Table 1:** Major dry eye causing agents.

**Causing agents**	**Molecular mechanism/impacts**	**References**
**Inflammation** (80%) cause of severe quantitative DES •**Graft vs. Host Disease** (GVHD) following transplantation. •**Autoimmune Diseases**, -Sjogren's syndrome (SS), Sjogren's syndrome Dry Eye (SSDE) and others, **-**Non-Sjogren's syndrome	***The onset of Lymphocyte trafficking***, -T-cell infiltrates and CD3, CD4, and CD8 Upregulation -CD11a and HLA-DR are lymphocyte activation markers.	(5)
**Age-dependent hyposecretion**, •Atrophy of the lacrimal gland tissue	**Age-related structural changes, e.g.**, -Atrophic acini, fibrosis, ductal obstruction, and lymphocytic infiltration to a high degree. **Functional changes, e.g.**, -Downregulated stimulated protein secretion	(6)
**Conjunctival inflammation**	Closure of the ducts and scarring	(5)
**Neurotrophic deficiency** •Congenital defect or trauma, •Infection (viral or bacterial).	Innervations dysfunction due to infections	(7)

The LG (serous tubuloacinar lacrimal gland) secretes fluids transported to the ocular surface *via* a small channel or duct system (1). The LG is also supported by two accessory secretory glands, the Krause and Wolfring glands (2). Tear fluid is a complex mixture of proteins, inorganic salts, and immunoglobulins such as IgA that can be studied (10, 11). LG parenchymal cells produce these components, which include acinar, duct, and myoepithelial cell types in a specialized structural arrangement forming distinct tubules of acinar, duct, and myoepithelial cells (12). Acinar cells (>80%) are the primary producers of lacrimal fluid, while duct cells (10–12%) play a minor role in the further modification of tear fluid compositions (13). Macrophages, plasma cells, lymphocytes, B cells, helper and suppressor T cells, dendritic cells, and mast cells are just a few of the cell types that reside in the interstitial space of the LG parenchyma. The tear film (secreted by meibomian or tarsal glands) consists of three layers: mucous (innermost layer, secreted by conjunctival goblet cells), intermediate aqueous layers (secreted by LG), and an outermost lipid layer (secreted by LG) (2–4). Tear film functions include lubrication and smoothing of the corneal epithelial layers, preservation of optic properties, moistening of the corneal/conjunctival epithelium layers, participation in metabolic activity, cleaning of the ocular surface by removing dust and debris, and prevention of microbial infections. Due to a lack of corneal epithelial stem cells complete loss of the aqueous layer might result in keratinization of the ocular surface epithelial cells and keratinocyte invasion from the skin (14, 15). A similar study has demonstrated the critical nature of tear film stability and the essential nature of preserving their complex compositions. LG is tightly regulated by the nerve systems (parasympathetic and sympathetic); as DES is produced by even modest changes in the composition and stability of the tear film (13).

There has been significant research for defining the various causing factors and Characterization of the molecular mechanism of different factors causing LG dysfunction and resulting in DES in human and animal models. In addition, other treatment strategies are also being developed by studying animal models and some human clinical data. Here we review the current information available about the various treatment modalities, animal models and human clinical data to treat and restore ocular surface disorders.

## Molecular mechanism of DES in animal models and humans

### Factors causing tear deficiency

Lacrimal gland impairment and consequent declined tear secretions are the most prominent reasons for DES. Studies in animal/humans derived information about the reasons showing age-dependent hyposecretion, inflammatory reactions causing LG destructions, conjunctival inflammation/scarring causing duct closures, enervation dysfunction because of congenital defects and trauma, any sort of pathogenic infection (bacterial/viral) causing LG dysfunctions. Aging and female gender are related to the higher risk for causing dry eyes in humans (female, age < 50, risk potential of dry eyes 5.7%; age > 45, risk potential 9.5%) (3). LG shows various age-dependent characteristics, including atrophy in acinar cells, fibrosis, obstruction of ducts, and lymphocyte infiltrations, are shown to be displayed by LG.

#### Age-dependent development of DES

Age-dependent DES is characterized by atrophic acini, fibrosis, ductal blockage, and an increasing prevalence of lymphocytic infiltration (6). Functional alterations in older mice, such as decreased triggered protein production, were found.

#### Inflammation

**Figure 2:**
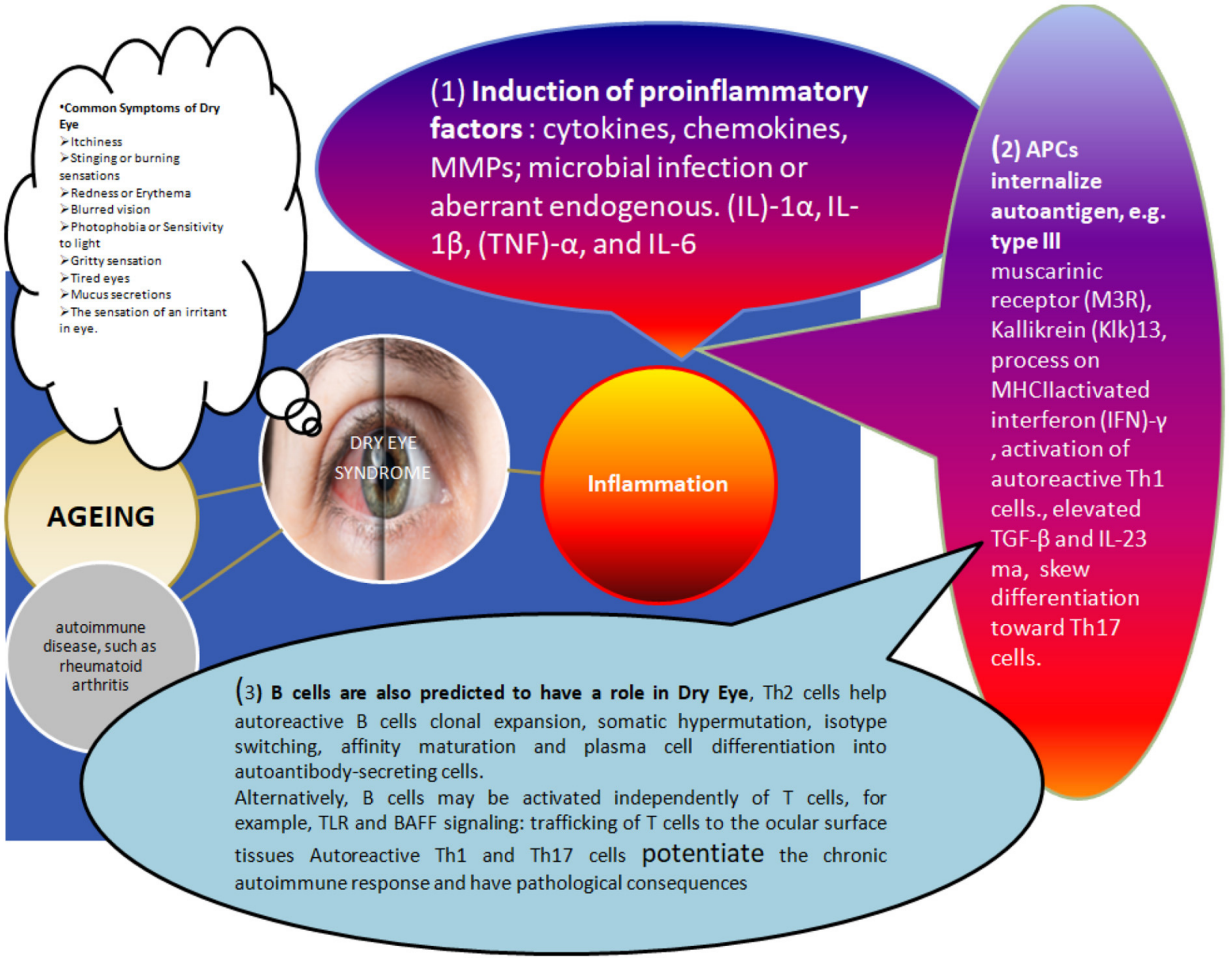
Different factors causing dry eye associated changes in the human ocular surface and disease manifestations: The immunopathogenesis of Dry Eye disease involves many causing agents, including age-related changes, Hormonal imbalance, and infections. Still, most of these agents predominantly display their impact through violation of the ocular surface's delicate immune-regulatory mechanism. The inflammatory response caused by various factors triggering these immune changes can be described as (1) Initiation of Induction of proinflammatory factors: as shown in the figure, cytokines, chemokines, and matrix metalloproteinases (these initial signals can trigger MMPs). This might involve microbial infection-induced downstream signaling [e.g., Toll-like receptor (TLR) signaling]. Various acute inflammatory cytokines (e.g., IL-1α, IL-1, tumor necrosis factor TNF- α, IL-6, etc.) are reported in higher concentrations in dry eye conditions and can amplify inflammatory reactions through APCs activation. In addition, APCs secreted IL-12 and INF-γ eventually activates the differentiation of autoreactive T-cells (e.g., Th1 cells). (2) Secondly, A DIFFERENT COMBINATION OF CYTOKINE AND THEIR ELEVATED CONC (e.g., IL-6, TGF-β, IL-23) can skew toward Th17 cells. Thirdly, B cells also contribute to all these inflammatory changes Related to dry eyes. For example, autoreactive B cells are triggered by Th2 cells helping their clonal expansion and activation of somatic hypermutation isotype switching, affinity maturation and ultimately differentiation of plasma cells into autoantibody-secreting cells (in addition, independent activation mechanisms also do exist, e.g., TLR and BAFF signaling pathways). Migration of autoreactive T cells: Adhesion molecules (e.g., LFA-1, VLA-4), chemokine receptors (e.g., CCR5 and CXCR3) and their interaction with the cognate receptors on ocular cell surface cells (e.g., ICAM-1, CCL5, and CXCL10) can direct the migration of these autoreactive T-cells. Further, Autoreactive Th1 and Th17 cells support chronic immunoresposes causing immunopathological consequences.

Both acute and chronic inflammation are shown to be the most common causative agent (>80%) for severe DES (Figure 2) (2). One of the major causing agents has been Graft vs. host diseases (GVHD) (40–76%) following the transplantations, and the severity of GVHD is directly reflected in the severity of DES (16). GVHD could induce lymphocytic infiltration of both accessory and lacrimal glands, followed by fibrosis of acini and ducts, declined aqueous tear component secretion due to accumulation of amorphous materials, cell debris along with normal-appearing granules (associated with age-associated tear deficiency markers) in the acini and ducts (17). On the other side, inflammation can result in a variety of consequences, including GVHD, other autoimmune disorders, including Sjögren's syndrome Dry Eye (SSDE), and non-syndrome Sjögren's Dry Eye (non-SSDE) (3, 4, 9, 18). Histological examinations of animal and human lacrimal gland biopsy samples (DES lacrimal gland) revealed the normal duct arrangement breakdown in LG, as evidenced by reduced lobular patterns, lymph follicle buildup, fibrosis, and atrophied acinar cells (Figure 2) (19). CD4+ T cells dominated the lymphocytic infiltration, with a lesser proportion of CD8+ T cells and B cells (20, 21). However, immunohistochemical studies revealed additional details about several lymphocytic activation markers, including HLAs, lymphocyte function-associated antigen, and very late antigen-4, as well as an increased level of IFN-ɤ (Interferon- ɤ), cell adhesion molecules (e.g., intracellular adhesion, vascular cellular adhesion molecule) on acinar, ductal, and epithelial cells in LG and conjunctiva (5, 21, 22). Proinflammatory cytokines such as IL-1 and IL-1 have been found in high concentrations in the tear fluid of DES patients. In some cases, they are also associated with the early stage of leukocyte invasion and corneal injury (23). Thus, DES may be described as a multifactorial condition resulting from trauma, age-related degeneration, inflammatory agents, and any pathogenic microbial infection of the LG, corneal surface, or other structures (24, 25). The complex tear system is tightly regulated by a delicate balance of these various factors. Slight changes in any one of them (altered tear fluid composition, decreased blinking frequency, resulting in decreased ocular surface lubrication) can trigger systemic inflammatory responses, which can result in inflammation of the lacrimal gland or other components of the LFU (24, 25).

## Modern treatment/management modalities for DES and related ocular complications

Most current clinical care techniques are palliative in nature. They remain reliant on therapeutic agents such as lubricating agents, tear substitutes, and tear retention, as well as the use of anti-inflammatory drugs to alleviate ocular surface inflammations (Table 2) (49). Additional techniques, such as partial or complete submandibular salivary gland transplantation, have demonstrated significant alleviation in a small number of patients (50–53). While the distinctive Schirmer test scores and extended tear breakup time (TBUT) are usually used to describe the severity of DES, the whole process does not affect tear film function or visual acuity in patients. One such explanation stems from the fact that the content of tears differs significantly from that of LG gland secretions. Salivary gland transplantation produces hypoosmolar liquids that cause microcystic corneal oedema and epithelial abnormalities (50). Additionally, xenotransplantation of swine lacrimal glands with architectural similarity to human lacrimal glands is presented as a viable therapy option with substantial rejection and retroviral infection risks (54–58).

**Table 2:** Therapeutic agents for dry eyes.

**Therapeutic agents**	**Mode of action**	**Impact**	**Limitations**	**References**
•**Cyclosporine A (**CsA): Fungal-derived peptide Trade name Tacrolimus: Restasis	-Block the transcription of cytokine genes in activated T cells -Inhibition of IL-I, -Inhibition of apoptosis by blocking the opening of the mitochondrial permeability transition pore (MPTP)	Increasing the density of conjunctival goblet cells; the hydrophobicity of non-aqueous ophthalmic vehicles, and their low aqueous solubility	Systemic administration: nephrotoxicity and hypertension Topical ocular delivery: poor intraocular penetration,	(26–28)
•**Corticosteroids** Fluorometholone and Loteprednol Etabonate	-Inhibiting MMPs (matrix metalloproteinases), - Inhibition of inflammatory cytokines and adhesion molecule production	- Highly reduced central corneal fluorescein staining scores are recorded & -Significant decrease in inferior bulbar conjunctival hyperemia can be recorded	-**NOT** suitable for long-term use due to side effects, inherent risks of high IOP, and Cataractogenesis	(29, 30)
•**Tetracycline derivatives:** Minocycline, Doxycycline	**Inhibition**/**reduced production of the followings**: MMPs (IL-I), Tumor necrosis factor (TN F)-alpha -Collagenase, phospholipase A2 -Bacterial flora producing lipolytic exoenzymes, -Lipase production, Anti-angiogenic effect	↓ Ocular surface symptoms, ↓ In meibomian lipid breakdown products, ↑ Clinical parameters in dry eye-associated diseases	↑ Risk of breast cancer and morbidity ↑ Inhibition of angiogenesis ↑ Effectiveness in rosacea-related disorders.	(31–34)
•**Autologous serum**	Lubrication contains other biochemical components that allow them to mimic natural tears and epitheliotrophic growth factors.	Not defined yet	Inconsistency in possible benefits, Dilution, Storage, Safety Exposure to contaminated SED should be avoided.	(35–37)
•**IL-Ra** Anakinra	-Competitively inhibit the binding of IL-1α and IL-1β to IL-1 receptor I, IL-1Ra suppresses IL-1–mediated inflammation	Can significantly improve corneal healing and reduce inflammatory damages	Still in the experimental, analytical phase and only animal studies are reported	(38, 39)
**Resolvin E1 (Rx-10001)** Endogenous immune response mediators η derived from the lipoxygenation of the essential dietary omega-3 polyunsaturated fatty acids, eicosapentaenoic acid, and docosahexaenoic acid	The agonists of resolution, pro-resolution lipid mediators (PRM), “stop” PMN and eosinophil infiltration, stimulate non-phlogistic recruitment of monocytes, enhance macrophage phagocytosis of apoptotic PMN, increase lymphatic removal of phagocytes, and stimulate mucosal antimicrobial defense.	Increased tear flow promotes a healthy epithelium, while decreased cyclooxygenase-2 Expression reverses dry eye damage to the corneal epithelium.	Under clinical trial	(40, 41)
•**Chemokine Receptor Antagonist** Monocyte chemotactic protein 1/chemokine receptor antagonist	Migration of chemokine receptor antagonist-bearing mononuclear cells is inhibited.	Compared to the vehicle-treated and untreated, dry eye groups, there was less infiltration of corneal CD11b(+) cells and conjunctival T cells.		(42)
•**Tofacitinib (CP-690,550)** Selective inhibitor of the Janus kinase (JAK).	Immune cell activation, proinflammatory cytokine production, and cytokine signaling are all inhibited.	IL-2, IL-4, IL-7, IL-9, IL-15, and IL-21 are among the cytokines that block signaling through the common chain containing receptors.	•Causes potential adverse reactions when administering long-term systemic therapy •The clinical trial indicated improving both signs (Schirmer's test without anesthesia and corneal fluorescein staining) and symptoms.	(43)
**SAR 1118** Investigational small-molecule lymphocyte function-associated antigen-1 antagonist	A critical step in T-cell activation (normal immune response and inflammation) is the binding of LFA-1 on the surface of T cells to intercellular adhesion molecule-1 on endothelial, epithelial, and antigen-presenting cells.	Effective inhibitor of T-cell activation, adhesion, migration, proliferation and cytokine Symptoms measured with OSDI (total ocular surface disease index) showed a significant improvement in corneal staining scores and visual-related improvements in the release.9	Under clinical trials	(44, 45)
•**Mapracorat** (Formerly ZK-245186 and subsequently BOL-303242-X) is a novel selective glucocorticoid receptor agonist	A novel selective glucocorticoid receptor agonist, anti-inflammatory	Hyperosmolar-induced cytokine release was inhibited with activity and potency comparable to that of a commonly used steroid.	Under investigation	(46, 47)
•**Additional nutritional supplements** e.g., essential fatty acids including omega-3, linoleic acid, and gamma-linoleic acid,	Anti-Inflammatory Properties Arachidonic acid and specific proinflammatory lipid mediators are formed from omega-6 fatty acids (PGE2 and LTB4). On the other hand, certain omega-3 fatty acids (such as EPA found in fish oil) inhibit the synthesis of these lipid mediators and the production of IL-1 and TNF-alpha.	Adjuvants for a dry eye treatment that have been proposed Topical treatment with alpha-linolenic acid significantly reduced corneal fluorescein staining in dry eye-induced animals compared to vehicle and untreated controls, decreased CD11b(+) cell number, corneal IL-1 and TNF- Expression, and conjunctival TNF- Expression.	Need more detailed studies to define the clinical benefits	(48)

## Lacrimal gland regeneration modalities

In the absence of any effective cure for most DES patients, alternative approaches such as regeneration of the lacrimal glands or transplantation of bioengineered lacrimal gland tissue are being explored by various researchers. They have been demonstrated to have high clinical potential. First, the lacrimal functional unit components' damage or loss of function, e.g., lacrimal gland, can be categorized as either partial or complete. In the “partial damaged” conditions, an obvious treatment strategy could be regenerative measures for the residual cells or tissue. These approaches are non-invasive, and the *in-situ* microenvironment may provide superior support than any intro culture settings or artificial growth conditions. In the second category, if no residual cell is available for regeneration, *in-vitro* reconstruction of lacrimal glands or bioengineered lacrimal gland is the only option which is being developed using the most advanced tissue engineering techniques.

### Stimulation of the regeneration of existing tissue

These approaches should restore tear volume, accurate tear composition, and anti-inflammatory qualities to protect lacrimal gland cells from additional harm, allowing gland tissue regeneration, remodeling, and functional recovery. No immunogenic problems should be associated with these techniques, indicating a possible role for autologous implants or the availability of HLA-matched donors. Additionally, the administration of these therapies should be sufficiently efficacious to be completed in a single try, reducing the need for repeated intervention. Clinical use can be ensured by adhering to simple and fast GMP-compliant processes. Additionally, these approaches must be highly efficient, which means that if cells are to be delivered to recipients, they must be capable of homing in significant numbers. This would ensure success and cost-effectiveness while also being free of harmful consequences.

Numerous techniques are being investigated to accomplish these goals, which can be classified as (i) Drugs and (ii) Cell-Based Therapies.

### Drugs or pharmacological agents for regenerative approaches

All previously known drugs for the Treatment of quantitative DES are only met for palliative relief and anti-inflammatory activities to reduce further damage to the ocular surface. For example, cyclosporine A, tacrolimus, and corticosteroids are routinely prescribed for reducing lacrimal gland inflammation (49). Another range of pharmacological agnates which can improve tear flow rates, such as lacritin, lactoferrin, quercetin, and systemic pilocarpine, are being investigated without showing any regenerative potential (59–65).

There are several intriguing reports describing the use of “autologous platelet-rich plasma” (PRP) injections and “tumor necrosis factor-stimulated gene/protein 6 (TSG-6)” for lacrimal gland regeneration (66, 67). PRP injection close to the lacrimal gland improved tear volume, increased the TBUT, and decreased ocular surface staining in DES patients. Numerous growth factors are contained in PRP, including epidermal growth factor, platelet-derived growth factor, insulin-like growth factor, and neural growth factor. In addition, VEGF is a vascular endothelial growth factor (68) that promotes cell migration, adhesion, proliferation, and differentiation in various cell types. Apart from its use in the treatment of a variety of diseases, such as wound healing, corneal ulcers, chemical burns, fibroblast proliferation, and extracellular matrix remodeling *via* collagen remodeling (69–73), PRP is an effective epithelial recovery of the cornea following laser-assisted *in situ* Although the mechanisms underlying PRP's regenerative effects are unknown, one possibility is that it induces epithelial-mesenchymal transition (EMT) cells, which are well-known for their critical roles in tissue repair, remodeling, and regeneration in a variety of glandular tissues, including mammary glands, liver, and lacrimal gland (67, 74). TSG-6's anti-inflammatory properties are further demonstrated in studies examining the regenerative and immunomodulatory functions of MSCs in several glandular tissues, including the skin, kidney, and liver, which identified TSG-6 secretion as a mechanism by which MSCs manifest their protective properties (63, 75, 76). Although the molecular mechanism and additional detailed investigations are required to establish their clinical utility, all of these reports showed a relatively easy and adaptable technology that might be developed for DES regenerative medicine.

### Cell-based regenerative approaches

A few findings highlight the importance of cell-based techniques as a potentially beneficial therapeutic strategy, as evidenced by research in humans with GVHD/SSDE and in animal models (77–83), Most of these research proved the utilization of mesenchymal stem cells to regenerate lacrimal or salivary glands. Freudenstein and colleagues described the *de novo* formation of osteogenic foci employing a bone marrow (BM) derived cell population termed MSCs for the first time in mice model experiments (84). Significant data has been generated since then defining MSCs surface identification markers (e.g., CD29, CD73, and CD105 positive; CD34 and CD45 negative), isolation procedures and culture standardizations, and their potential therapeutic roles in a variety of tissue types in different animal models and humans (85–87). MSCs, for example, can be classified as dormant multipotent or adult stem cells that exist in an undifferentiated condition in most adult organs and tissues, alongside fully differentiated cells (88).

MSCs have been shown to differentiate into mature muscular (85), corneal epithelial (86), salivary acinar (89), pancreatic and neural cells (85, 86).

MSCs are derived from various sources and have been demonstrated to possess anti-inflammatory capabilities. MSCs suppressed the maturation/proliferation of a variety of immune cells [e.g., T-cell, B-cell, natural killer (NK) cell, monocyte, macrophage, and dendritic cell]; they can impair B-cell Ig synthesis and reduce NK cell cytokine release (90). MSC's anti-inflammatory characteristics suggested they could also be used therapeutically in chronic inflammatory ocular surface illnesses.

MSCs have been explored for their therapeutic potential in salivary and lacrimal gland damage models (Table 3). For example, injection of IL-1α directly into the mouse lacrimal gland has been shown to elicit acinar cell death, resulting in a transient drop of tears and a subsequent regenerative response (92). Additional studies demonstrated the presence of MSC-like cells (nestin- and vimentin-positive cells) that may be responsible for these regenerations in IL-1-treated animals (74, 92, 93). These indirect shreds of evidence, combined with their adipogenic differentiation properties and ability to survive *in vitro* beyond 14 days, indicated that IL-1 injection may have triggered the destruction of the lacrimal gland, which could be recovered by MSC or other stem progenitor cells in these animal model studies.

**Table 3:** Cell-based therapies for lacrimal gland regeneration.

**Type of cells/conditioned medium**	**Mode of experiment and impact**	**References**
Induced Pluripotent stem cells (iPS) -derived conditioned medium	Injection in mouse Tail-vein 1h before irradiation; Partially-improved tear secretion	(91)
Injection of xenogeneic human MSCs (hMSCs) into the periorbital space of an inflammation concanavalin induced dry eye mouse model	10^3^-10^5^ Hmsc per mouse eye injected after causing inflammation Restored tear secretion (1 week post concanavalin injection)—reduced T cell inflammation	(82)
Injection of allogeneic MSCs into the area surrounding the main lacrimal gland of dogs with naturally occurring DES	Used 8 × 10^6^ hMSC/dog eye Schirmer test score restored to with normal range. Ocular discharge reduced -hyperemia reduced –corneal changes reduced	(80)
Topical administration of Autologous MSC to the corneas of rats following benzalkonium Chloride to induce DES	7 × 10^5^ MSC per rate Schirmer Test Score Increased -TBUT increased -corneal defects reduced	(78)
Intravenous (IV) injection of allogeneic MSC to patients with DES secondary to GVHD	1 × 10^6^ MSC/kg -Schirmer test score increased -TBUT increased -corneal defects reduced	(81)
Salivary gland, Tail-vein injection of allogeneic BM-MSC into a mice model of SS		

Subsequent research has bolstered the putative role of MSC in IL-1 or other injury-induced tear insufficiency models. Numerous gene expression markers, including Nestin, Musashi-1, ABCG2, Pax6, Chx10, and Sox2, putative stem cell markers nestin and α-smooth muscle actin (SMA), or ΔNp6, Nanog, Oct4, Sox2, and Pax6, transcription factors (CMyc and Kruppel-like factor-4), and early lineage markers for endodermal (GATA4, GATA6) and mesodermal (bone morphogenic proteins-4 and−7), have been reported (86, 94).

#### MSCs-based therapies and *in vitro* studies

MSCs' therapeutic activities have also been established in various *in vitro* investigations conducted by diverse research organizations. MSCs' well-known ability to secrete growth factors [e.g., epidermal growth factor (EGF), transforming growth factor (TGF), and vascular endothelial growth factor] is thought to support their growth-inducing effect in a variety of tissues such as the corneal limbus, kidney, lung, and skin. Growth factors [e.g., epidermal growth factor (EGF), transforming growth factor (TGF), and vascular endothelial growth factor] Caplan et al. (95), Chen et al. (96), Akram et al. (97), Hu et al. (98), Moghadasali et al. (99), and Walter et al. (100). Similarly, MSC-conditioned media obtained from mouse lacrimal glands enhances the migration and proliferation of porcine lacrimal gland epithelial cells *in vitro* (101). Numerous animal models studied for DES demonstrated beneficial effects of MSC transplantation or topical application, including the mouse model, the rat model (benzalkonium derived chloride), dogs, and other canine models, resulting in improved Schirmer test scores and TBUT increment, decreased corneal defects, sustained decreased ocular discharge, hyperemia, and corneal changes over time (77, 79, 80, 83).

Although direct evidence for MSC-derived therapy in human dry eye patients is scarce, some studies exist. For example, a small study (22 patients with GVHD-related dry eyes) indicated that intravenous infusion of human MSCs (BM-derived) improved Schirmer test scores and decreased ocular surface disease index scores in some recipients (81). Although elevated levels of IL-2 and IFN-γ were observed in individuals who had beneficial benefits following MSC treatment in these investigations, this finding contradicts previously documented MSC involvement in lowering these proinflammatory cytokines in dye eye models.

It shows that MSCs are potentially valuable for developing lacrimal gland regenerative strategies. Still, the complex relationship between different immunogenic environments and their effect on MSC-derived therapeutic needs to be defined at a deeper molecular level. Second, identification and culturing methods to obtain large amounts of MSC for these clinical uses will also be another area of research.

### Other alternatives: Human amniotic membrane (hAM) epithelial cells and induced pluripotent stem (iPS) cells in cell therapies for dry eyes

Both human amniotic membrane (hAM) epithelial cells and iPSCs are being investigated for salivary gland regeneration in animal models, indicating the possibility of using them for lacrimal gland regeneration as well, owing to the structural and functional similarities between the salivary and lacrimal glands in mammals (102). This is further corroborated by results demonstrating that hAM epithelial cells can be transdifferentiated into acinar cells in an *in vitro* double-chamber system research (103). Additionally, iPSCs may be of enormous regenerative value in techniques for lacrimal gland regeneration, but no direct trial evaluating their potency has been reported so far. However, studios that use iPS cell-conditioned media to regenerate defective mouse lacrimal glands are available, demonstrating their potential anti-inflammatory qualities that may be useful in lacrimal gland regeneration (91). However, numerous elements of their effects, efficacy, and manufacturing for clinical use have yet to be rigorously explored to establish an acceptable regular treatment alternative for dry eye syndrome.

### Gene therapy for DES

This is another promising approach for several incurable disorders, including regenerative medicine. The target molecules (i.e., DNA, mRNA, mi RNA, siRNA, antisense oligonucleotides etc., for any gene sequence) are delivered through various delivery approaches such as viral and non-viral methods and analyzed for the desired impact to achieve potential therapeutic effects. The major obstacle remains the use of optimal delivery approaches in clinics. While viral particle-based strategies are effective and specific, they have a high risk of viral infection. While non-viral vectors are safer than viral vectors, they currently have low delivery efficiency (104).

Nevertheless, *in vitro* and *in vivo* investigations have revealed the possibility of creating anti-inflammatory medicinal solutions for lacrimal glands. For example, in an autoimmune rabbit model, blocking tumor necrosis factor-α by transferring an inhibitor gene resulted in normal tear formation, increased TBUT and rose bengal scores, and decreased lymphocyte infiltration (T-cell, CD18+cells) (105). Thomas et al. demonstrated similar results employing adeno-associated virus (AAV) vector-mediated viral (v) IL-10 gene expression in the lacrimal gland (LG) immunopathology and ocular surface disease (rabbit model) (106).

## *In vitro* manufacture of lacrimal gland tissue and transplantation strategies

**Figure 3:**
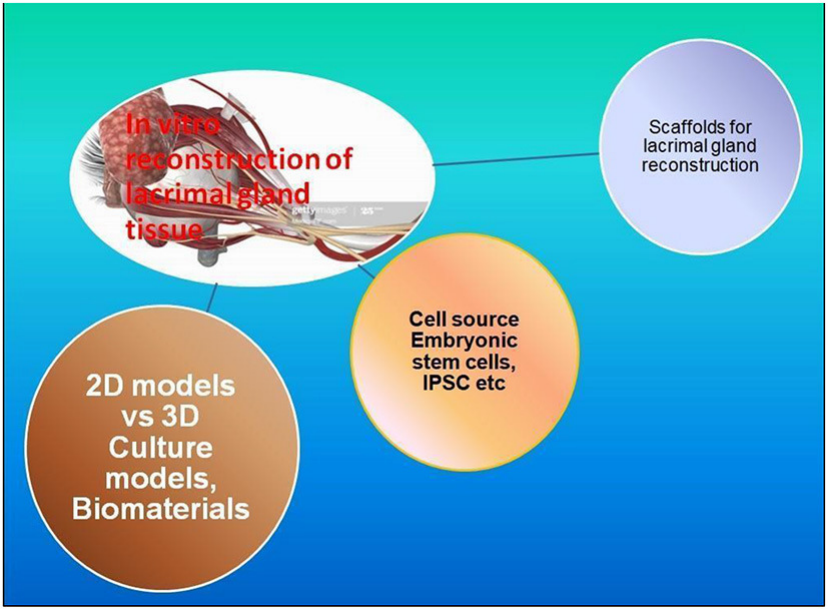
Components of *in vitro* reconstruction of Lacrimal glands: there are many efforts reported to develop strategies for the *in vitro* culture of clinical-grade lacrimal glands/tissue. The whole idea revolves around (1) Cell sources and Types; Adult stem cells, ESCs, IPSCs, or MSCs; (2) Biomaterials and their suitability for tissue reconstruction assessment through 2D/3D *in vitro* culture models; (3) Scaffolds architecture, fabrications and culture associated biochemical, physical and functional parameter assessment.

Another important approach is being explored when there are no resident lacrimal gland cells, as discussed above, that remain to be triggered for regeneration. In these situations, *ex vivo* construction of the lacrimal glands using tissue engineering techniques could be the only solution, as discussed here. To ensure the functionality of these *ex vivo* generated tissue, several complex approaches would be required to define the set of parameters, including, 1. Tear volume and composition. 2. Heterogeneity of the cells in tissue-cultured, e.g., acinar epithelial, endothelial, and duct cells. 3. No adverse effects and non-immunogenicity. 4. Mimic a niche environment to sustain the long-term functionality of fully differentiated cell types in the transplant. 5. Three-dimensional or native-like the structural organization of the implant. 6. Optimal vascularization. 7. Optimal Innervation and cannulation to ensure secretion and supply of lacrimal fluid in response to various cues and their transport to the ocular surface for tear film generation. 8. A broad range of criteria applicable to their *in vitro* mechanical handling, Quality assessment, biomaterials used for easy approval from regulatory authorities and cost-effectiveness (Figure 3).

### Cell source for bioengineered lacrimal gland generation approaches

Various cell sources have been successfully demonstrated in *ex vivo* tissue engineering approaches, such as Embryonic stem cells, iPSC or Epithelial progenitor cells. The lacrimal gland is a complex organ where different cells such as lacrimal acinar cells, myoepithelial cells, and duct cells are required to coexist in a functional interconnected architecture to ensure their functional roles in tear production secretion and transport. As discussed in the following sections, different cells from Allogeneic, autologous or Xenogenic sources are being explored for the regular GMP-compliant lacrimal gland implants. may be xeno-, allo-, or autologous; embryonic stem (ES) cells; or induced pluripotent stem (iPS) cells.

### Scaffolds and biomaterials for *ex vivo* bioengineered lacrimal gland reconstruction

Scaffolds play essential roles in various organs' *ex vivo* tissue engineering approaches. They offer several benefits in achieving the goal of organ construction and their implantations. For instance, firstly, the *Total number of cells* permitted for the transplantations, i.e., a large number of viable cells should be transplanted to restore complete functions of the glands or other organs. Scaffolds facilitate this by providing a suitable platform for many cells to grow simultaneously in a controlled microenvironment. Secondly, Scaffold may help in the process by **providing a surface** on which each cell type can grow and *in situ* guided growing location for each cell type may be dictated to help achieve functionality. In addition, the precisely selected physicochemical properties of biomaterials (regulating mechanical strength or degradation rates, etc.) may be chosen and designed to enhance the specific application of functions of the maturing tissue, e.g., lacrimal gland.

Biomaterials are essential for constructing these scaffolds, and various of them have been described with variable properties. Therefore, one can select between Natural or synthetic biomaterials depending upon the priorities. Natural biomaterials are advantageous due to biomimicry but are immunogenic and might not be available in sufficient quantities for some time. On the other hand, biosynthetic biomaterials are a rapidly evolving field inheriting several advantages and can be used easily to develop Good Manufacturing Practice (GMP)-compliant products/methods.

### Advancements in the bioengineering strategies for lacrimal glands by 3D/2D culture of lacrimal cells

Generation of the functionally active lacrimal gland might require many cells (e.g., lacrimal epithelial cells or stem cells etc.) to be seeded in 3D scaffolds or similar 3D culturing approaches. Since 2D culture systems are comparatively more straightforward and may be helpful in the Characterization and assessment of functionally relevant markers, activities etc., they are most often used before the actual 3D environment could be established. In addition, the various quantitative and qualitative parameters studied by 2D culture experiments are further useful in achieving the GMP-compliant 3D culture protocols and their validation before any clinical application could even be hypothesized. There are several 2D culture studies reported using different species (i.e., rat, mouse, rabbit, and human) to demonstrate adult lacrimal epithelial cells isolation, culture methods, Media formulations, and other growth environments using 2D culture methods (107–116). These studies established the requirement of FCS and other growth factors for *in vitro* culture media formulation, including epidermal growth factor, hepatocyte growth factor, insulin-like growth factor and fibroblast growth factors. However, recently serum-free culture environments such as Hepato-STIM without or reduced FCS concentration have shown clinical suitability (113, 117).

### Embryonic stem cells and induced pluripotent stem cells and lacrimal gland generation

Embryonic stem cells are pluripotent stem cells with enormous research and clinical significance. The potential transcriptional factors were identified in a direct differentiation method for the lacrimal gland generated by using human ESC, resulting in differentiation into LG epithelial cell phenotype (118). These studies demonstrated the potential use of Pax6, CK15, AQP5, and lactoferrin as an inducer for lacrimal gland generation. This can be achieved further by developing a stable gene expression model system for these factors and ESC. There is substantial clinical potential for ESC, but its use is restricted due to the associated risk of tumor formations and ethical concerns (119). Hu-iPSC was demonstrated as an alternative to these ESC-associated ethical concerns, and they are being explored for various tissue regeneration and reconstruction strategies. However, no report shows lacrimal gland cell generation using iPSC as source materials. Direct differentiation of iPSC into lacrimal gland cells is still open, offering enormous opportunities for researchers and clinicians to develop effective methods for the large-scale production methods of lacrimal gland dysfunctions in the near future.

#### Lacrimal gland reconstruction studies and characterization of cell cultures by 2D methods

The simplicity of 2D cell cultures offered the researcher an opportunity to demonstrate various cellular and culture-associated characteristics for the various components that can be useful in defining the qualitative and quantitative parameters of their functional significance in more complex organs or *in vivo* studies. Many different methods, such as Electron microscopy (both scanning and transmission) (107, 108, 110–114) has been a valuable method to identify showing secretory granules, the polarity of these cells intercellular contacts which can be used to examine the types and nature of these cell-cell interactions in a more complex 3D culture system. Similarly, many immunohistochemical analyses in 2D culture systems could help identify non-epithelial and myoepithelial markers. For instance, the immunohistological examination could recognize the Expression of cytokeratin 3/12, E-cadherin (non-epithelial markers), and distinct cell population expression epithelial cell adhesion molecule, p63,30 pan-cytokeratin (pan-CK) etc. showing their potential for quick assessment approaches (109, 116, 120). In addition, there are other essential markers which could also be determined by these studies, such as aquaporin5 (AQP5, CK822), myoepithelial (α-smooth muscle actin) cell marker lacrimal gland-specific protein secretion (117, 121), Further studies have been done to measure; β-hexosaminidase activity as a result of parasympathetic stimulation of these 2D culture methods (110, 112–114, 122).

### Biomaterials and various approaches to culture lacrimal gland cells

Several materials have been tested in 2D/3D culture methods to exemplify the growth conditions for more complex organ constructions in a 3D culture system. These materials might be useful in developing scaffolds, thus providing specific molecular signals or physicochemical cures that can trigger cellular growth and differentiation under controlled environments. Some of the most studied biomaterials may include Polyethersulfone tubing, Poly (L-lactic acid), Polyester, Human amniotic membrane, and Matrigel. Unfortunately, there are few reports about each material and its potential application for developing robust culturing protocols for clinical uses. In the following segment, we discuss a few experiments using these materials and their outcomes to understand their further technical uses.

#### Approached for developing scaffold using polyethersulfone, poly (L-lactic acid) and polyester

Polyethersulfone (PES) is a well-known synthetic polymeric material for its exceptional biological compatibility (i.e., oxidative, thermal, and hydrolytic stability) and its mechanical and film-forming capabilities. PES membranes are commonly used in biomedical equipment such as blood purifiers (hemodialysis, hemofiltration, and hemodiafiltration), plasma separation (123), and hemodiafiltration (124–128). These widespread applications demonstrated the materials' good blood compatibility, and efforts might be made to construct immune-protective devices utilizing hollow fiber membranes containing xenologous pancreatic islets for subcutaneous implantation in diabetic rat models (129). Subsequently, it was demonstrated that PES hollow fibers might support the growth of a variety of human cells *in vitro* as a substrate; for example, human endothelial cells kept their morphological and functional properties when grown on fibronectin-coated PES hollow fibers (130, 131). These studies provided an essential basis for exploring PES-based scaffolds for culturing lacrimal epithelial cells. Using a PES scaffold could support as a substrate for cellular growth and immune protectant by physically prohibiting immune cells of the host cell from reaching the graft/cells growing inside these fibers/tubing but does not inhibit the release of lacrimal fluids from them. For instance, PES tubes (50 mm length × 3 mmdiameter, dead-end) seeded with rat lacrimal gland acinar cells were successfully cultured exhibiting permeability of the small molecular components (e.g., glucose) while non-permeable to large molecules such as IgG antibodies, etc. (108). These studies indicated possibilities for the use of PES based scaffold for further lacrimal gland cell culture. However, their clinical use would only be possible after the rigorous evaluation of all relevant information about the growth and function, such as lacrimal fluid volume and composition in human and animal models.

#### Poly (L-lactic acid)

It is also well-known material investigated by making scaffolds in thin (20 μm) membrane form, also in collaboration with collagen type I, silicone, and Thermanox coverslips (114, 122). The Scaffold designed by these researchers showed a thriving culture of rabbit lacrimal acinar cells and the excretion of small molecules. Still, it did not allow the permeation of IgG-like giant molecules in culture. However, cells were reported to grow to a sub-confluence level with suboptimal functionality levels as described by these reports (122).

#### Polyester

It has also been evaluated in the form of microporous polyester cell culture inserts (122). Authors in these studies revealed the relationship between autoimmune disease conditions caused acinar cell dystrophy and hypothesized a possible molecular mechanism for functional quiescence of M3 muscarinic acetylcholine receptors due to agonistic auto-antibodies mediated inhibition of downstream signaling mediators such as Gq and G11 in lacrimal parenchymal cells (132). The report indicated the establishment of a confluent cell monolayer (3–5 days of culture) of acinar epithelial cells on polyester membrane Transwell inserts. This study also described the transepithelial electrophysiological behavior of cultured cells using the classic “Using short-circuit methods.”

The cultured cells were positive for tight junction membrane marker “occludin” and showed similar electrophysiological behavior as indicated by their responsiveness to carbachol stimulation and measured increased β-hexosaminidase activity (released by rabbit lacrimal acinar cells in response to cholinergic stimulation) in these studies. These studies indicated a potential use for this biomaterial in lacrimal gland reconstruction, which would require more intense research in the 3D environment.

### Human amniotic membrane as a substrate to culture lacrimal gland cells

The placenta's innermost layer, often named Amniotic membrane Using (AM), has been widely used for various types of cell culture as an excellent extracellular matrix support material. AM can be described as a three-layered membranous structure consisting of the epithelium, basement membrane (T = thick layer), and stroma layer (avascular). Several reports state the supportive role of AM in cell adhesion, migration, proliferation, and differentiation of epithelial cells (133–135). Human AM is already used for ocular surface regeneration showing anti-inflammatory and non-immunogenic qualities. Since it is already demonstrated in human experimentations, it is more attractive from the regulatory issues related point of view in clinical trials (136, 137). Schrader et al. (113) demonstrated small lacrimal cell cluster (2–5 cells) generation containing characteristic intracellular granules with the highest secretion capability after 3 days of *in vitro* culture (113). The author reported a sustainable culture and formation of a more lacrimal gland-like structure (containing lumen) for the next 2–3 weeks, and the sigh of necrosis and some spindle-shaped cells (de-differentiation indicator) started becoming visible in superficial cell layers. These effects were explained as a result of dilution of growth factors from hAM, and/or there may be a decline in the close physical contacts between cells of lacrimal origin (superficial layer) and basement membrane cell proteins from hAM. These results highlighted the need for close cell-cell and cell-to-substrate contact during *in vitro* reconstruction protocols. Alternatively, it is proposed that the poorly vascularized structure of hAM may not be sufficient to support neovascularization and may be unable to support acinar growth causing necrosis. Further, the hAM is 2D culture since cells can only be seeded on the superficial surface. Thus, the membrane may not be exemplified as a 3D scaffold culture system requiring many cells to grow and differentiate in a more complex architectural arrangement.

### Matrigel for lacrimal gland reconstruction

Matrigel is widely used for various research experiments, including lacrimal gland reconstruction. The material contains many basement membrane proteins (e.g., laminin, collagen IV) and growth factors (e.g., TGF-β, FGF, EGF, IGF) (138, 139). Matrigel has been demonstrated to be an excellent growth medium with collagen type I which can support lacrimal cell viability for up to 30 days (with or without 3T3 cells). The cells start forming clusters quickly on the 2nd day of culture and can form an acinar-like structure with high efficiency (117). Studies using collagen only (with or without 3T3 cells) were not capable of supporting cellular viability similar to Matrigel, showing superior qualities of the later substrate. These excellent growth supportive properties over other substrates such as hAM were also further confirmed for the lacrimal and other glands (120, 140). These studies indicate that selecting individual substrates for large numbers of cells for reconstruction purposes would be a significant step. Using Matrigel would be less problematic as long as pre-expansion of lacrimal cells is concerned. Although the clinical application and approval for that might have to demonstrate the safety issue more rigorously since Matrigel is mouse sarcoma-derived, there are hidden risks such as tumorigenesis, which should be avoided at any cost. Similar human tissue derivatives could emerge in the future, providing excellent clinical applications and acceptability at the medical and ethical levels.

### Approaches using simple 3D cultures for lacrimal gland reconstruction

#### Rotary cell culture systems (RCCS)

The method was originally developed for the assessment of cell and tissue culture possibilities in space or in the absence of gravitational forces (141) and later proven useful in supporting tissue structure development, providing essential cell-cell contacts, and showing a potential culture approach with high mass transfer and low shear forces (142, 143). Schrader et al. (112) demonstrated 28 days of culture of Lacrimal gland acinar cells (rabbits) in an RCCS showing the formation of acinar-like cell conglomerates (spheroids) when assessed by electron microscopy after the 7th day of culture. They were optimally functional till day 14, which was indicated by the appearance of a central lumen structure filled with secretory material. The Electron microscopic analysis of the Cells after 7 days of culture revealed polarity with a nucleus at the base and electron-lucent and electron-dense secretory granules in the apical regions, microvilli, and well-connected apical cell parts through tight junctions or desmosomes. At this stage, the overall size was measured to be ~384.6 μm (111.8) in diameter, and a tiny fraction (24.4%) of the spheroids were shown to have apoptotic cells with a condensed small nucleus. The culture reached an optimal state on the 14th day, {size: 382.4 (92.2) μm.} when Acinus-like structures were frequently visible, and the central luminal area was filled with secretory materials inside the spheroids. However, the size and frequency of viable acinar cells started decreasing after the 21st day [290.7 (62.8) μm], and viable cells were visible only in the peripheral regions. Still, large centers of apoptotic cells appeared in 20% of the spheroids. This apoptotic death continued further and reached up to 41.7% of the spheroids, causing a substantial size reduction [388.9 (179.2) μm] on the 28th day of culture. Nevertheless, viable cells were still visible in the peripheral region of the spheroids.

Throughout these time intervals, acinar cells could be detected secretory in response to carbachol stimulation (a parasympathetic stimulant) reduced over the culture period. The gradual increases in the apoptotic activities in the spheroidal central regions might indicate reduced nutritional supplies. The possible explanation could be their non-vascularization, Posing a limitation of these spheroidal cultures for clinical transplantations. Any improvement in the design to trigger vascularization of these spheroids may be of greater use from a clinical point of view.

### Matrigel in 3D culture setting for lacrimal glands

The previous section discusses Matrigel as an excellent medium for various cells in 2D culture systems, including lacrimal acinar cells. Matrigel in 3D Scaffold was also demonstrated to have similar growth supporting characteristics in the “raft cultures” approach (110). This method was developed by improvising the previously described use of Matrigel-coated plastic plated methods hypothesizing a more *in situ* culture averment would be available for lacrimal growth assessment in these studies. The culture was observed for up to 28 days, while acinar cell cultures started appearing on 5–7 days surrounding a central lumen-like structure. The regular culture evaluation for secretory components and prolactin etc., along with other morphological assessment criteria (as mentioned in different sections of the article), indicated a similar growth pattern with optimal growth between 7 and 14 days of culture, which started diminishing beyond 21 days of culture and reached to the minimal level on 28th day of the culture assessment. These studies indicated superior culturing qualities for Matrigel over other culture materials. However, the non-suitability of Matrigel due to its animal origin still makes it less attractive for clinical transplantations.

### Decellularized xenogeneic tissue for lacrimal gland reconstruction

Using decellularized tissue for reseeding cells and further regeneration has been extensively studied. It offers many advantages over synthetic materials-based scaffold approaches. It provides precise membrane protein composition, accurate 3D architecture allowing secretion of secretory fluids, adequate vascularization, innervations, and many more benefits. There are reports using decellularized porcine lacrimal gland (3 mm diameter) and lacrimal acinar cells for lacrimal gland regeneration/reconstruction (144). The authors' used domestic pig (*Sus Scrofa*) derived lacrimal gland tissue and prepared decellularized LG scaffold (1% sodium deoxycholate and DNAse solution) for these studies (145, 146). Since complete decellularization is essential to avoid any immune rejection, as confirmed in these studies, no significant changes were reported in the composition and architecture of the Scaffold (basement membrane) etc. Subsequently, cells were reseeded in the decellularized matrix, and proper epithelial cell growth and cell polarization were evident, showing characteristic acinar-like structures formation by these cells as described in other studies. As demonstrated in these studies, the cells seeded on the decellularized Scaffold formed a multilayer tissue harboring epithelial phenotypic characteristics after the 7th day of reseeding culture. Subsequently, their migration started, and a duct-sphere-like structure was observed.

Further identification of mucous in the cytoplasm and the presence of secretory vesicles confirmed their functionality as a lacrimal gland acinar cell/tissue. In addition, the secretion function of lacrimal gland acinar cells was observed for up to 30 days. The method shows great promise and must be studied further to scale up the number of cells (which would be required for the human recipients). More specific seeding, such as laser-guided reseeding, could further improve by exploring effective and efficient reseeding methods for the ductal and vascular structure to improve the output. Since xenobiotic tissue implantation in humans is already established, this provides a higher opportunity for regulatory agencies' approval for its clinical use (57). Similarly, the bovine ductal matrix (decellularized) is successfully demonstrated to reconstruct a lacrimal gland duct. This further supports the feasibility of using the same xenobiotic strategies for LFU component reconstruction for clinical use (147).

## 3D co-culture of adult lacrimal gland cells along with progenitor-like cells

### Culture in the absence of scaffolds

Lacrimal progenitor cells are evident in normal and injured tissues, which could be exploited to give rise to a functional lacrimal gland under an appropriate 3D culture environment (86, 92, 93, 148, 149). The immature or less differentiated lacrimal gland epithelial cells (human, ABCG2+ and c-kit+ mixed population) can develop into a sphere and duct-like tissue structures when the culture lacks any scaffold (120). Co-culture of mixed differentiated and undifferentiated lacrimal cell cells has been subjected to develop “microspheres,” which could be useful in increasing the potent stem cell/progenitor cells in these *in vitro* culture procedures for further applications [(150) abstract] (151). This co-culture approach for lacrimal epithelial cells, along with other specific cell types in any 3D culture technique, would better support the *in situ* microenvironment, and their corresponding interactions might help in mimicking and therefore maintaining similar phenotypic interactions of each cell type present *in vivo* (152).

### Culture with scaffolds

Culturing the mixed lacrimal epithelial cells (both differentiated and undifferentiated progenitor cells) from a rabbit model on a low adherent plastic have been demonstrated to spontaneously generate lacrimal gland cell spheres [(150) abstract]. The Matrigel-coated decellularized lacrimal glands were used as a scaffold, and lacrispheres-derived cells discussed above were used for reseeding them in a 3D culture format. An immunohistochemical assay performed to define their cellular composition afterwards indicated the existence of (both differentiated) acinar and ductal epithelial cells and myoepithelial cells/progenitor-like cells as indicated by the Expression of pan-cytokeratin, c-kit, lactoferrin, and α-smooth muscle actin markers.

The β-hexosaminidase activity was observed as a measure of functional activity, which remains visible up to 17 days after seeding. The parasympathetic functional activities described here were maxed on 7–10 days and diminished after the 17th day of culture. The functionality was also confirmed by assessing mRNA level HEXB gene expression, which was reported to be increasing till day 14 and then started declining, indicating a beneficial impact of the inclusion of undifferentiated epithelial cells since, unlike all previously reported assays, the functional activity remain detectable even after culture assay was timed out.

## Summary and conclusion

The tear film is vital for ocular surface health and visual acuity and a variety of components, including lacrimal glands, meibomian glands, goblet glands, accessory secretory Krause and Wolfring glands; conjunctiva epithelial cells and corneal epithelium are collectively responsible for the production, secretion, and regulation of the tears in normal human healthy eyes. Many different causing agents are now defined as damaging factors that perturb delicate homeostasis in human eyes. Most frequently mentioned causing agents and defining markers for ocular surface health are linked to inflammations that may lead to cytological and molecular level changes in the ocular surface tissue.

Despite decades of research and understanding of the disease etiology, clinical management remains palliative, and many different types of lubricating agents/strategies, including artificial tears substitutes, Gels/Ointments, Moisture chamber spectacles etc., are the only available treatment. Advanced treatment approaches may include Anti-Inflammatory Therapy which has been demonstrated to use pharmacological agents such as Cyclosporine, Corticosteroids, Tetracyclines, etc., autologous serum, and similar approaches. However, none of these approaches has eliminated the disease. Most of the time, they are useful in managing the acute inflammatory associated situation and relieving the pain in the patients. On the other hand, cell-based approaches seem to be comparatively new and promising but need to be explored in much detail before they can offer a long-term solution to the problem. One of the crucial methods demonstrated in these years is partial or total submandibular salivary gland transplantation, which has shown remarkable success in some recipients but suffers from many inherent limitations, such as no improvement in the tear film health and visual acuity. Another similar approach that needs attention here could be xenotransplantation of porcine lacrimal glands, which is still in the developmental phase and possesses high risks of retroviral infections and graft rejections.

Most advancements in the medicinal fields include regenerative approaches and tissue engineering strategies which are being developed for ocular surface regeneration and reconstructions by using a variety of cell types and culturing fabrication protocols. Regenerative medicine involves studying the defining markers and procedures to identify the residual stem cells population, which can be triggered to regenerate and recover the tissue's lost functional capabilities, such as lacrimal glands. Regenerative remedies can be defined both as those which are based on ”Pharmacological Drugs” such as cyclosporine A, tacrolimus, corticosteroids, lacritin, lactoferrin, quercetin, systemic pilocarpine, “*autologous platelet-rich plasma” (PRP) injection, and “tumor necrosis factor* α*-stimulated gene/protein 6 (TSG-6),”* etc. that are being investigated for their impact on the various clinical symptoms of DES.

On the other hand, “Cells Based Approaches” such as MSCs are the most frequently demonstrated cells due to their immense regenerative potential reported for various other organs and tissue in clinics. MSCs are reported to have remarkable anti-inflammatory properties against immune cells (e.g., T-cell, B-cell, natural killer cell monocyte, macrophage, and dendritic cell). They can decline Ig production and release cytokines from immune cells. This way, MSC can help by reducing inflammatory damages to the tissue and improving its growth/repairing. This is evident in studies defining MSC secret growth factors [e.g., epidermal growth factor (EGF), transforming growth factor-β (TGF-β), and vascular endothelial growth factor] that induce cell proliferation and angiogenesis in wound healing. However, any direct evidence of MSC in DES treatments is still awaited. Present information based on MSC in animal models and other *in vitro* studies are also required to define more elaborated mechanisms related to the issue. In addition, there would be a requirement for a large amount of GMP-compliant cells for the regular clinical use of MSC. That requires an established process of regular, reliable, and reproducible ways of culturing them to yield sufficient numbers of cells.

Similar evidence for human amniotic membrane (hAM) epithelial cells and iPSC shows their curative, regenerative potential through *in vitro* and animal model-based studies. However, no direct evidence shows any clinical report in humans. Further, the GMP-compliant culturing protocols and other related issues are a rate-limiting step in most cell-based regenerative therapies.

Another promising area of therapeutics being explored includes Gene therapy. This approach may require the identification of specific gene/protein targets, which may be manipulated by altering their Expression or interactions etc., by delivering specific small molecules (i.e., DNA, mRNA, mi RNA, siRNA, antisense oligonucleotides). The success of therapeutics is regulated mainly by the efficacy of the delivery vehicles and their ability to avoid off-target impacts. There is some progress in the *in vitro* and animal model studies showing therapeutic potential for many anti-inflammatory gene expression regulatory strategies. But the experimentations on human recipients are yet to be defined. Altogether these reports indicate massive progress in lacrimal gland regeneration medicine approaches, and more clinical studies would pave the way to extend these preliminary studies beyond their present state of experiments to the clinics.

When the resident cells with enough reproducing potential cannot be found, “implantation of *ex vivo* manufactured cells,” tissue or organs are the most probable choice. *In vitro* manufacturing of lacrimal gland and other ophthalmic tissue are also an expanding area of research. Standard 2D culturing and 3D culture procedures, biomaterials, and fabrication of specific scaffolds are already demonstrated for different lacrimal epithelial cells. As discussed in the main text, various specific parameters such as tear volume, tear compositions, functional efficacy, degree of innervations in implanted tissue, etc., remain crucial while evaluating these strategies, fabrication process, cultivation method, or product itself before it could be implicated in any experimental or clinical settings. Various types of cells, including ESCs, iPSC, and Epithelial progenitor cells, are being explored for manufacturing clinical-grade ophthalmic tissue, bioengineered lacrimal acinar cells/gland, cornea, and many more. ESC and IPSC are well-established for their differential potential in any type of tissue and organ if specific culture conditions/microenvironment are provided along with architectural guidelines to define their structure and shape-related (mechano-physicochemical parameters) details. Defining their mechanochemical and biochemical characteristics might be essential for any growing tissue/organoids such as lacrimal gland, and scaffolds designed by following these characteristics in mind are vital for the successful generation of these implants. Biomaterials are required to manufacture these scaffolds, and natural and synthetic biomaterials are well-studied in 2D/3D culture settings. Since 2D culture systems are comparatively more straightforward and may be helpful in the Characterization and assessment of functionally relevant markers, activities etc., they are most often used before the actual 3D environment could be established. Thus, various growth condition parameters, growth factors (e.g., epidermal growth factor, hepatocyte growth factor, insulin-like growth factor and fibroblast growth factors; w/+ FCS or w/o FCS), and intracellular molecular mechanisms define the regulatory mechanism (e.g., Pax6, CK15, AQP5, and lactoferrin) of this differentiation could be established by using 2D culture methods and ESC/IPSC cells as source materials. Scaffolds can be fabricated by using either naturally occurring biomaterials such as collagen etc. or may be manufactured by using synthetic polymeric chemicals including Polyethersulfone (e.g., tubing), Poly(L-lactic acid), polyester, etc., Human amniotic membrane and most famous Matrigel which is evident to have extraordinary growth supporting properties. Matrigel seems to be the most promising biomaterial from growth and differentiation supporting properties as described through enormous reports. However, there may be some controversies regarding the use of Matrigel in clinical settings due to its animal-based origin. Polyethersulfone (PES) based scaffolds are useful in generating rat lacrimal glands as described in the main text but remain to be demonstrated for their similar efficacy with human cells. PES-scaffolds would also require their rigorous evaluation for generating sufficient tear volume and adequate tear compositions before they could be taken as a standard method of choice for *ex vivo* lacrimal gland generation. Similar studies using Poly (L-lactic acid) and Polyester-based scaffolds and culturing the animal cells for lacrimal glands have been highly promising. Still, they might require further standardization and optimization with human cells as demanded by PES-scaffolds.

Another critical approach reported the use of microporous polyester-based cell culture inserts, which were impressive while demonstrating the transepithelial electrophysiological behavior of cultured cells, which could be an essential tool in defining the natural-like functionality of manufacturing implants.

In short, manufacturing lacrimal gland tissue could be feasible with the advent of appropriate culturing procedures, Scaffold generating methods, biomaterials, and rigorous biological assessment for various relevant parameters as described herein near future. Both 2D and 3D culturing techniques would remain useful in determining multiple *in vitro*/*in vivo* cultures associated and functionally relevant parameters, which would pave the way for the generation of optimal *ex vivo* manufacturing process and production along with post-implantation assessment would be possible to eradicate the DES associated ocular surface disorders in patients.

## Author contributions

VS: conceptualization, writing, drafting, editing, and final decision. PS and UV: editing, drafting, and upgradation. RC: conceptualization and specific discussion suggestions.

## Funding

SERB - Empowerment and Equity Opportunities for Excellence in Science, Project No (EEQ/2019/000422).

## Conflict of interest

Author VS reports grants from SERB during the study: as generated by ICMJE.org. The remaining authors declare that the research was conducted in the absence of any commercial or financial relationships that could be construed as a potential conflict of interest.

## Publisher's note

All claims expressed in this article are solely those of the authors and do not necessarily represent those of their affiliated organizations, or those of the publisher, the editors and the reviewers. Any product that may be evaluated in this article, or claim that may be made by its manufacturer, is not guaranteed or endorsed by the publisher.
